# Can BioSAXS detect ultrastructural changes of antifungal compounds in *Candida albicans*?–an exploratory study

**DOI:** 10.3389/fphar.2023.1141785

**Published:** 2023-07-18

**Authors:** Kai Hilpert, Christoph Rumancev, Jurnorain Gani, Dominic W. P. Collis, Paula Matilde Lopez-Perez, Vasil M. Garamus, Ralf Mikut, Axel Rosenhahn

**Affiliations:** ^1^ Institute of Infection and Immunology, St. George’s, University of London (SGUL), London, United Kingdom; ^2^ Laboratory Analytical Chemistry—Biointerfaces, Ruhr-University Bochum, Bochum, Germany; ^3^ TiKa Diagnostics Ltd., London, United Kingdom; ^4^ Helmholtz-Zentrum Hereon, Geesthacht, Germany; ^5^ Institute for Automation and Applied Informatics (IAI), Karlsruhe Institute of Technology (KIT), Karlsruhe, Germany

**Keywords:** *Candida albicans*, antifungal peptides, antimicrobial peptides, BioSAXS, SAXS (small-angle X-ray scattering), mode of action (MOA)

## Abstract

The opportunistic yeast *Candida albicans* is the most common cause of candidiasis. With only four classes of antifungal drugs on the market, resistance is becoming a problem in the treatment of fungal infections, especially in immunocompromised patients. The development of novel antifungal drugs with different modes of action is urgent. In 2016, we developed a groundbreaking new medium-throughput method to distinguish the effects of antibacterial agents. Using small-angle X-ray scattering for biological samples (BioSAXS), it is now possible to screen hundreds of new antibacterial compounds and select those with the highest probability for a novel mode of action. However, yeast (eukaryotic) cells are highly structured compared to bacteria. The fundamental question to answer was if the ultrastructural changes induced by the action of an antifungal drug can be detected even when most structures in the cell stay unchanged. In this exploratory work, BioSAXS was used to measure the ultrastructural changes of *C. albicans* that were directly or indirectly induced by antifungal compounds. For this, the well-characterized antifungal drug Flucytosine was used. BioSAXS measurements were performed on the synchrotron P12 BioSAXS beamline, EMBL (DESY, Hamburg) on treated and untreated yeast *C. albicans*. BioSAXS curves were analysed using principal component analysis (PCA). The PCA showed that Flucytosine-treated and untreated yeast were separated. Based on that success further measurements were performed on five antifungal peptides {1. Cecropin A-melittin hybrid [CA (1–7) M (2–9)], KWKLFKKIGAVLKVL; 2. Lasioglossin LL-III, VNWKKILGKIIKVVK; 3. Mastoparan M, INLKAIAALAKKLL; 4. Bmkn2, FIGAIARLLSKIFGKR; and 5. optP7, KRRVRWIIW}. The ultrastructural changes of *C. albicans* indicate that the peptides may have different modes of action compared to Flucytosine as well as to each other, except for the Cecropin A-melittin hybrid [CA (1–7) M (2–9)] and optP7, showing very similar effects on *C. albicans*. This very first study demonstrates that BioSAXS shows promise to be used for antifungal drug development. However, this first study has limitations and further experiments are necessary to establish this application.

## Introduction

Similar to the problem of antimicrobial resistance in bacteria, human pathogenic yeast and fungi also show an increase in resistance (Bhattacharya et al., 2020; Kean and Ramage, 2019; Murray et al., 2022). With the growing numbers of immunocompromised patients, the resistance of opportunistic fungi to antifungal drugs is of concern since only four classes of antifungals are available for treatment. For example, patients who are undergoing cancer treatment (chemotherapy), have transplants, have diabetes, or are infected by the human deficiency virus (HIV-1/2) are at higher risk of getting infected by fungi, especially yeast. In addition, immunocompetent individuals can also experience symptoms of yeast overgrowth, for example, topical, oral, vulvovaginal, and intra-abdominal, causing estimated annual cases of more than 130 million (Arastehfar et al., 2019). The most common cause of candidiasis is the opportunistic yeast *Candida albicans*.

Small-angle X-ray scattering (SAXS) is a powerful tool to study ultrastructures and their changes in macromolecules and nanomaterials in solution or suspension (Boldon et al., 2015; Honecker et al., 2022; Sartori and Marmiroli, 2022; Sun et al., 2022; Byer et al., 2023). The combination of a short wavelength and a large penetration depth of the photons used allows the investigation of large volumes that contain millions of objects in less than a second, providing unique analytical possibilities (Franke and Svergun, 2020). That allows for gathering structural information on disordered systems and, due to the large penetration depth, resolving the inner structures of such systems. After the sample has been irradiated, the scattered photons are recorded with a spatially resolved 2D detector.

In our pioneering work over the last years, we have shown that the ultrastructural changes caused by the treatment of bacteria with classical antibiotics and novel antibacterial compounds can be detected by BioSAXS (Von Gundlach et al., 2016a; 2016b; 2019; Hilpert et al., 2021; Rumancev et al., 2022). In this approach, BioSAXS is used as a structurally sensitive fingerprint method that reveals subtle differences at the cell wall or in the intracellular structure of the pathogens, which are caused by the drug treatment directly or as a response of the microorganism towards the assault by the drug (Rumancev et al., 2022). The intracellular structural changes occur on different length scales, and a combination of SAXS and ultra small-angle X-ray scattering (USAXS) allowed to separate contributions from at least four different structure sizes, which were connected to major bacterial properties such as the shape or size of the ribosomes (Von Gundlach et al., 2016b). Recently, it was shown that also neutron scattering can provide additional useful information about the bacterial ultrastructure (Semeraro et al., 2022). BioSAXS can be used for Gram-positive as well as Gram-negative bacteria (Von Gundlach et al., 2019). In general, the differences in the scattering curves could be analysed using a principal component analysis (PCA), and plots of PCA 1 over PCA 2 can be used to visualise the results. We could demonstrate that distances between data points in those plots for untreated versus treated with different antibiotics correlated with their different modes of action (Von Gundlach et al., 2016a). However, it was unclear if BioSAXS could be used to support the development of urgently needed new antifungal drugs. Fungi are true eukaryotes and therefore are immensely more structured than eukaryotes. These various structures will be picked up by BioSAXS, and the question we address in this study is whether ultrastructural changes caused by the antifungal drug can be detected above the background of all signals stemming from existing structures within the cell. The focus of this study was exploratory and fundamental: can BioSAXS be used to discriminate between untreated yeast (*C. albicans*) cells and cells treated with an antifungal compound?

Flucytosine was used as a well-known antifungal drug. Flucytosine can be deaminated in yeast, but not in mammalian cells, hence its specificity and usage as a drug. The deaminated version will be incorporated into RNA and inhibit protein biosynthesis. In a further enzymatic reaction by uridine monophosphate pyrophosphorylase, 5-fluorodeoxyuridine monophosphate is produced. This compound inhibits thymidylate synthetase, and consequently, DNA synthesis is inhibited, also leading to further disruption in protein synthesis (Vermes et al., 2000). The antifungal compound was incubated with *C. albicans* for 40 min and then chemically fixed. SAXS curves were acquired and analysed by principal component analysis.

## Materials and methods

### Peptides

Antimicrobial peptides were produced by automated solid-phase peptide synthesis (SPPS) on a MultiPep RSI peptide synthesizer (Intavis, Tuebingen; Germany) and purified to the homogeneity of >90% by liquid chromatography-electrospray ionisation mass spectrometry (LC-ESI-MS, Shimadzu LC2020 system, Shimadzu, Milton Keynes, United Kingdom). For more details please refer to (Grimsey et al., 2020).

### Yeast strain

In this project, we used a clinical isolate of *C. albicans* obtained from Tim Planche (St. George’s, University of London).

### Bacteriological media and culture conditions

Mueller-Hinton broth (MHb) (Merck, Life Science UK Limited, Dorset, UK) was prepared to concur with the manufacturer’s directions and supplemented with 2% glucose to grow *C. albicans*. Liquid cultures were incubated at 30°C for 18–20 h on a shaker, and culture on solid media was incubated for 18–24 h at 30°C.

### Minimal inhibitory concentration determination

When it comes to determining the antimicrobial activity of antimicrobial peptides (AMPs) there are unfortunately many different approaches reported. Many AMPs are negatively affected by positively charged ions and other components of growth media, therefore Mueller-Hinton broth (MHb) is frequently used since it supports the growth of many organisms and seems to interfere little with the activity of most AMPs. The mode of action of Flucytosine and antifungal peptides were determined using a protocol (option E) of the highly cited publication by Wiegand et al. (2008) optimized for the work with antimicrobial peptides. This protocol uses MHb (0.2% beef extract, 1.75% casein hydrolysate). To adopt that media for the growth of yeast 2% glucose was added. For comparison, YPD complex medium (1% yeast extract, 2% peptone, 2% dextrose) is often used for the growth of yeast. Where this media provides the best activity for antimicrobial peptides the results should be carefully used when comparing MIC values to other antifungals measured in different growth media. A broth microdilution assay was applied using 6 × 10^5^ CFU/mL at 30°C. MICs were determined by the unaided eye, as the concentration in which no growth was observed.

### Sample preparation, measurement and data evaluation for BioSAXS

A detailed description of the sample preparation for BioSAXS, the BioSAXS measurement and consequently the data evaluation methods can be found in the open-source publication by Von Gundlach et al. (2019). Briefly, an aliquot of an overnight culture of a clinical isolate of *C. albicans* was diluted in Mueller-Hinton broth (MHb) and incubated for 2.5 h at 30°C on a shaker (225 rpm) to achieve log phase growth. The *C. albicans* culture was adjusted to have 1.2 × 10^6^ CFU/mL and aliquoted. The aliquots were then mixed with either sterile water or antifungal solution (2xMIC) to provide 6 × 10^5^ CFU/mL aliquots. Those were further incubated for 40 min at 30°C at 225 rpm. All aliquots were then centrifuged at 10,000 rpm and washed 3 times in ml 0.1 M PIPES [piperazine-N,N′-bis(2-ethanesulfonic acid)] buffer, pH 7.0. After washing, the pellet was resuspended in 1 mL of 2.5% glutaraldehyde v/v in PIPES buffer and fixated for 1 h at room temperature at 225 rpm. Consequently, the samples were washed 3 times with PBS buffer and resuspended in 100 µL of PBS and stored at 5°C.

The SAXS measurements were performed on the P12 BioSAXS beamline, EMBL (DESY, Hamburg), using a double crystal Si (111) monochromator. An energy of 10 keV with a 10^13^ s^−1^ photon flux was used. 30 μL of each sample was injected into the measuring chamber by an automatic sample robot (Arinax, BioSAXS), the focus of the X-ray beam was 0.2 × 0.12 mm. The scattering signal was recorded with a Pilatus 2 M detector (Dectris, Switzerland). For each sample, 20 measurements were made with 0.05 s exposure. Before and after each measurement the background was measured and automatically subtracted from the SAXS curves. Data analysis and evaluation were done using the open-source software SciXMiner (“Peptide Extension”) (Mikut, 2010; Mikut et al., 2017). To determine differences in the scattering curves between treated and untreated samples as well as between the different treatments a principal component analysis (PCA) was applied to the scattering curves. The data are measurements of about 100,000 cells that were measured 20 times to obtain the scattering curves. SAXS curves were averaged and the standard deviation was used as an error bar. The PCA analysis was carried out in the frequency range between 0.05–0.4 nm^−1^, and errors were propagated from the single SAXS curves (see Supplementary Figure S1).

## Results

**FIGURE 1:**
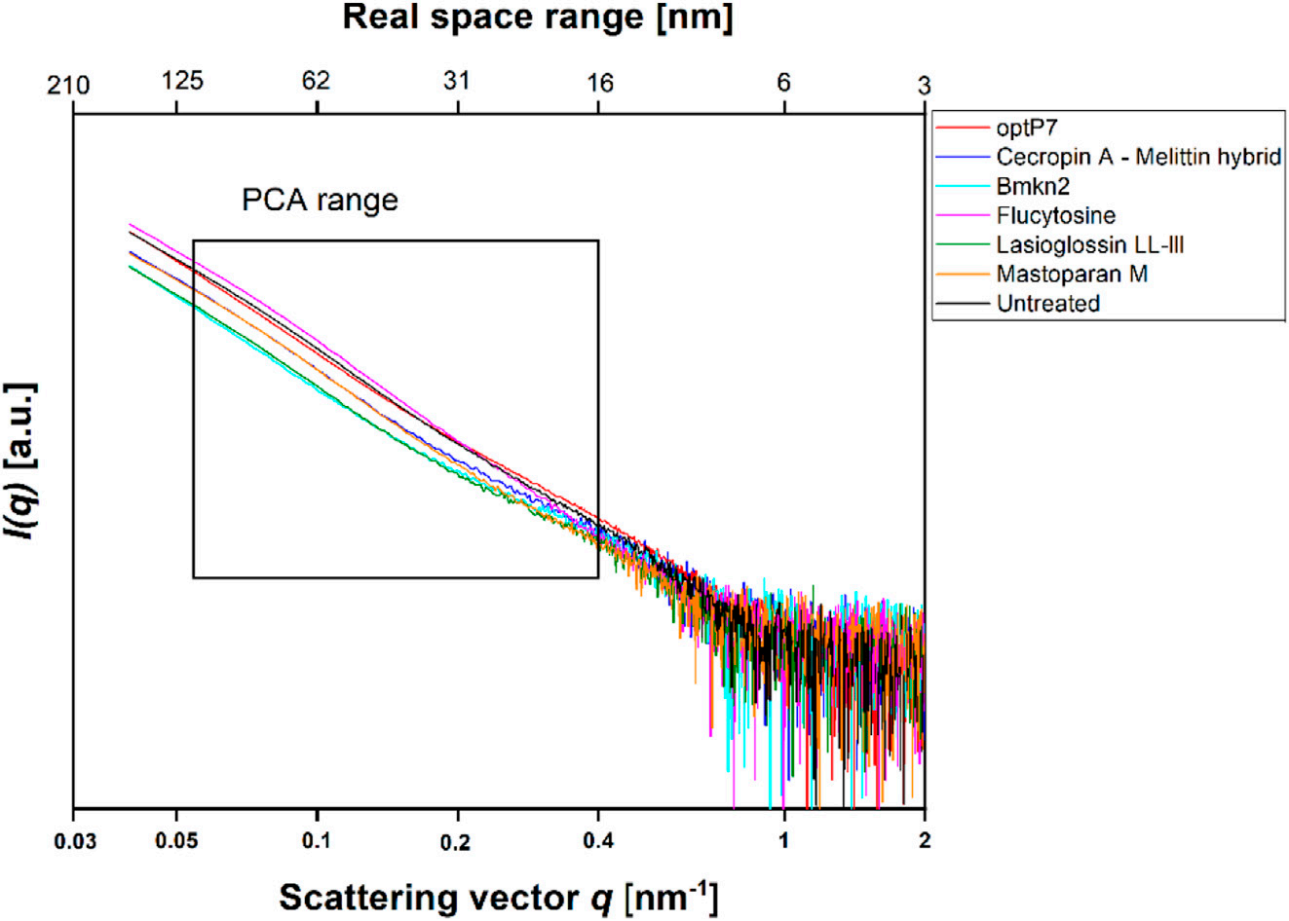
Small angle X-ray scattering data from *Candida albicans* as measured at the P12 BioSAXS beamline at PETRA III (Hamburg, Germany) at a photon energy of 10 keV. Untreated *C. albicans* as well as treated (40 min, 2xMIC) with antifungal peptides and Flucytosine were measured. The box indicates the range that was used to calculate the principal component analysis (PCA).

In this project, our main objective was to test whether BioSAXS can discriminate between untreated *C. albicans* cells and those treated with Flucytosine. The MIC of Flucytosine was determined to be 8 μg/mL. For bacterial culture, we found that 1 × 10^8^ CFU/mL is optimal for getting enough scattering signal to meaningfully analyse the curves from the BioSAXS experiment (Von Gundlach et al., 2016a). *Candida albicans,* however, is a eukaryote that, in its yeast form is about 5–10 µm in diameter, whereas, for example, *Staphylococcus aureus* is only 0.5–1.0 µm in diameter. In addition, eukaryotic cells are more structured and therefore may have a stronger scattering signal in a BioSAXS experiment than prokaryotic cells. Consequently, for the first experiments, we used only 6 × 10^5^ CFU/mL and combined this with 2xMIC for Flucytosine. Cells were incubated for 40 min and then fixated. Fortunately, the scattering curves showed good signal strength and could be used for further analysis. The scattering curves are presented in Figure 1.

**FIGURE 2:**
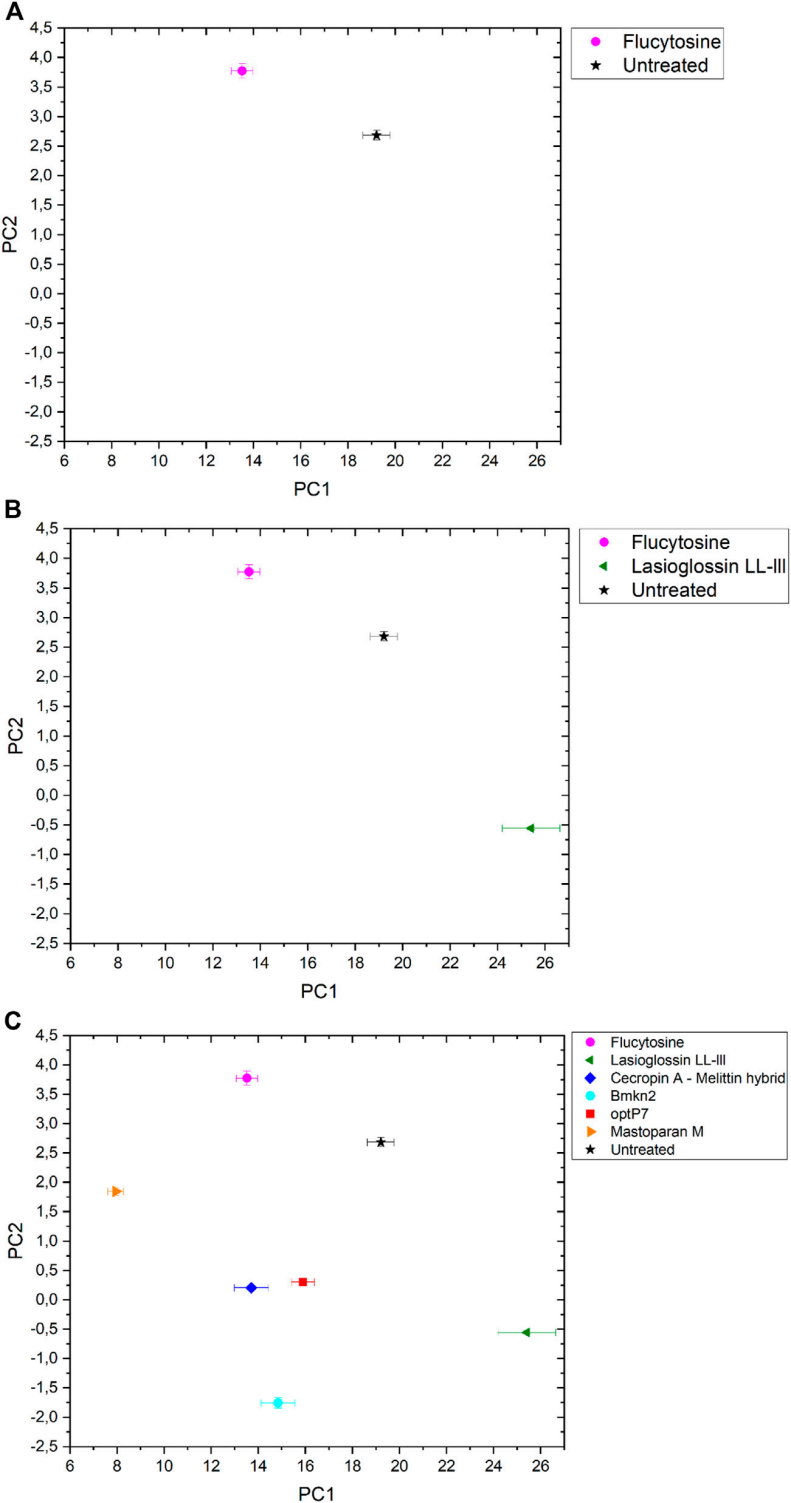
The linear coefficients of the first two principal components (PC) discriminate morphological changes and modes of action. **(A)** Flucytosine and untreated, **(B)** Flucytosine, Lasioglossin LL-III and untreated and **(C)** Flucytosine, Lasioglossin and various antifungal peptides. Error bars are standard deviations, propagated from SAXS curves.

The data was analysed by open-source data mining MATLAB^®^ Toolbox SciXMiner, using the “Peptide Extension” tool (Mikut, 2010; Mikut et al., 2017). A principal component analysis was performed to extract differences between the curves and for visual presentation, the values of the linear coefficients for the first two principal components were plotted, see Figure 2A.

The data are measurements of about 100,000 cells that were measured 20 times to obtain the scattering curve. In consequence, the data has high statistical robustness. SAXS curves were averaged and the standard deviation was used as an error bar. The PCA analysis was carried out in the frequency range between 0.05–0.4 nm^−1^, and errors were propagated from the single SAXS curves (see Supplementary Figure S1). Flucytosine induced ultrastructural changes after 40 min that in consequence separated well from the untreated sample. It shows a reduction of PC1 by about 6 units and an increase of PC2 by about 1 unit. This was the first time demonstrating that BioSAXS can be used to investigate antifungal compounds directly in yeast. That opens the way for further systematic studies similar to those done for antibacterial compounds on different bacteria. The main objective of this study was successfully fulfilled. Based on that success, a few antifungal peptides were selected for a further experiment.

Antimicrobial peptides are a class of compounds found in all four kingdoms of life that act against bacteria, fungi, viruses, and parasites. There are various databases for antimicrobial peptides, for example, APD, CAMP and DRAMP and DBAASP (Wang et al., 2016; Kang et al., 2019; Pirtskhalava et al., 2021; Gawde et al., 2023). DBAASP, for example, currently lists 5,595 antifungal peptides (Pirtskhalava et al., 2021). There is a huge potential to develop novel antifungals using these peptides as templates. Peptide libraries on cellulose can be used to investigate this large pool of compounds economically (Mania et al., 2010; Ashby et al., 2014; 2017; Knappe et al., 2016; López-Pérez et al., 2017). Of special interest are peptides that demonstrate a different mode of action than conventional antifungal drugs. Unfortunately, screening even small numbers of peptides to determine their mode of action is an enormous task and a great financial burden. Therefore, we decided to test one antifungal peptide (Lasioglossin LL-III) in a BioSAXS experiment. Lasioglossin LL-III was isolated from the venom of the eusocial bee, *Lasioglossum laticeps* (Cerovsky et al., 2009). The peptide displays favourable features for further drug development, including strong antimicrobial activity combined with low hemolytic and cytotoxic activity. The peptide can interact with charged membranes in a non-disruptive manner, depolarizes the membrane, and binds to DNA, suggesting DNA as an intracellular target (Battista et al., 2021). The peptide was able to kill various yeasts, including *C. albicans,* within 15 min and was shown to depolarize the plasma membrane (Kodedová and Sychrová, 2016). The antifungal activity of Lasioglossin LL-III against *C. albicans* was determined at 11 µM using a low inoculum of 1.2–7.5 × 10^3^ CFU per well (Slaninová et al., 2011). In addition, it was shown that the peptide inhibited temperature-induced morphotype changes toward hyphae and pseudohyphae (Vrablikova et al., 2017). The authors also showed that the peptide is an inhibitory agent in the DBA/2 murine model of vulvovaginal candidiasis and therefore a potential new drug candidate. The minimal inhibitory concentration (MIC) was determined using 6 × 10^5^ CFU/mL at 30°C to be 36 µM (Table 1). At two times the MIC and an incubation time of 40 min, *C. albicans* samples were prepared and measured by BioSAXS. The results are presented in Figure 2B. Surprisingly, there was a strong separation between the effects of Flucocytosin and Lasioglossin LL-III. Whereas Flucytosine acts on protein synthesis by RNA modification and also on DNA synthesis, Lasioglossin LL-III permeabilizes the membrane and interacts rather unspecifically with all DNA in the cell. BioSAXS measurements show that the ultrastructural changes are different. Especially PC1 is increased by 6 units compared to the untreated cells. In consequence, Flucytosine and Lasioglossin LL-III are separated by 12 units at the PC1 level. The PC2 value for the peptide is decreased compared to the untreated cells, whereas for Flucytosine it increased.

**TABLE 1:** Minimum inhibitory concentrations (MIC) in µM. Data are for *n* = 3 with modal values reported; all values were determined in Mueller-Hinton broth (MHb) supplemented with 2% glucose using 6 × 10^5^ CFU/ml *C. albicans*. All peptides are C-terminally amidated.

Peptide	Sequence	Origin	MIC (µM)	Literature
Cecropin A—melittin hybrid [CA (1–7) M (2–9)]	KWKLFKKIGAVLKVL	Designed	18	Andreu et al. (1992)
Lasioglossin LL-III	VNWKKILGKIIKVVK	*Lasioglossum laticeps* (Eusocial Bee)	36	Cerovsky et al., 2009
Mastoparan M	INLKAIAALAKKLL	*Vespa mandarinia* (Japanese wasp)	22	Hirai et al. (1981)
Bmkn2	FIGAIARLLSKIFGKR	*Mesobuthus martensii* (Manchurian scorpion)	18	Zeng et al. (2004)
OptP7	KRRVRWIIW	Computer designed	24	Grimsey et al. (2020)

As data obtained on the well-described Flucytosine and Lasioglossin was very encouraging, four less-studied peptides were analyzed and *C. albicans* cells treated with these compounds were measured by BioSAXS. The 16-mer peptide Bmkn2 was identified in the scorpion venom of *Buthus martensii Karsch* (Zeng et al., 2004). The peptide displays strong activity against gram-positive and gram-negative bacteria, however, this peptide is strong hemolytic with an HC_50_ of 17.13 μg/mL (Cao et al., 2012; Almaaytah and Albalas, 2014). Mastoparan M is a 14-mer cationic peptide that was isolated from the venom of the wasp *Vespa mandarinia* (Hirai et al., 1981)*.* It is active against gram-positive and gram-negative bacteria and shows very little hemolytic activity. In addition, it shows pro-inflammatory activity as well as anticancer activity (Wu et al., 1999; Wu and Li, 1999). The hybrid peptide from Cecropin A from *Hyalophora cecropia* (giant silk moth) and Melittin from *Apis mellifera* (European honey bee), called CA (1–7) M (2–9) has strong antibacterial activity against gram-positive and gram-negative bacteria, follow-up research shows that it is also active against anaerobe species and shows low hemolytic activity (>500 μg/mL) against sheep erythrocytes (Hultmark et al., 1980; Hultmark et al., 1982; Andreu et al., 1992; Edlund et al., 1998; Raghuraman and Chattopadhyay, 2007; Chetty et al., 2022). The peptide optP7 was obtained from a previously reported peptide library containing 3,000 members to better understand short antimicrobial peptides (Mikut et al., 2016). Based on the analysis of this data we developed a novel prediction method (unpublished results) for AMPs with low hemolytic activity. One selected candidate with strong antimicrobial activity and low hemolytic activity was the peptide optP7 (HC_50_ > 250 μg/mL against human erythrocytes). The peptide optP7 was selected for studying lipidation and glycosylation (Grimsey et al., 2020), cyclisation, and grafting into a cyclotide (Koehbach et al., 2021) that could be further optimized by procedures described previously (Hilpert et al., 2005; 2006; Mardirossian et al., 2018; 2019; 2020). In addition, the peptide was used to create hybrid peptides with improved activity and BioSAXS analysis showed different ultrastructural changes compared to both parental peptides (Hilpert et al., 2021). The MICs of all peptides are given in Table 1.

Flucytosine induced distinct different ultrastructural changes in *C. albicans* compared to all other antifungal peptides measured (see Figure 2C). It shows the highest PC2 value in the set and has the second-lowest PC1 value. Its specific way of interfering with RNA, DNA, and protein synthesis leads to changes in the cells that do not occur with the treatment of the yeast with the five peptides tested. The highest PC-1 value and the second-lowest PC-2- value is seen in Lasioglossin LL-III. The peptide Lasioglossin LL-III from the eusocial bee is therefore also well separated from all other antifungal compounds tested. Its ability to bind unspecifically to DNA and interfere with replication and RNA synthesis seems to induce rather unique ultrastructural changes. The lowest PC2 value was observed with the peptide Bmkn2 from scorpion venom. The lowest PC1 value was observed with the peptide Mastoparan M from the Japanese wasp. The 15-mer peptide Cecropin A-melittin hybrid [CA (1–7) M (2–9)] and the 9-mer peptide optP7 show very similar ultrastructural changes after 40 min of treatment. In contrast to the other peptides, they are both designed or computer-predicted. Short AMPs like optP7 have been shown to bind to ATP and inhibit ATP-dependent processes (Hilpert et al., 2010) and, in addition, depolarize bacterial membranes (Hilpert et al., 2006). Comparing *C. albicans* treated with all antifungal compounds versus the untreated sample, the differences between PC1 (ΔPC1) and PC2 (ΔPC2) can be calculated. Only Flucytosine shows a positive ΔPC1, demonstrating the different mechanism in comparison to the peptides. Interestingly, only Lasioglossin LL-III has a positive ΔPC1. In all other cases ΔPC1 and ΔPC2 are negative.

## Discussion

**FIGURE 3:**
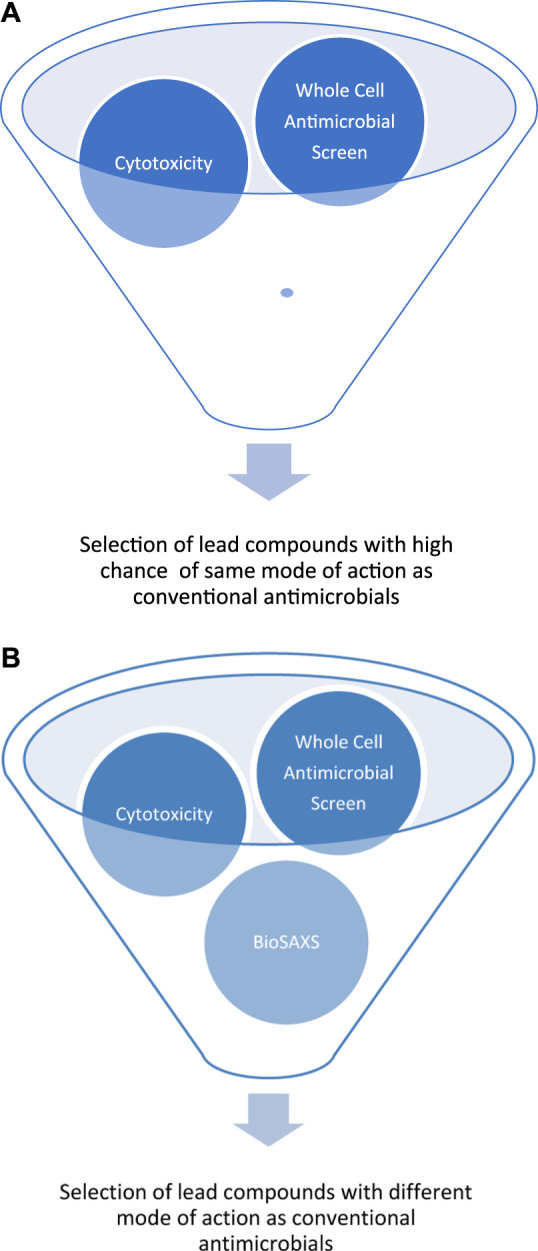
Schematic representation of early drug discovery process using a large compounds library against a targeted microorganism by a whole-cell screen, without BioSAXS, **(A)** and with BioSAXS, **(B)**. Taken from (Rumancev et al., 2022).

The development of resistance against antifungal drugs threatens successful treatment. Novel antifungal substances with new modes of action are urgently needed. Determining the mode of action is still time- and resource-consuming, so screening is not possible. Because of our pioneering work on BioSAXS on bacteria, we demonstrated that screening for novel modes of action is now possible. However, prokaryotes are not very structured in comparison to eukaryotes. In light of the presence of additional structures in eukaryotes, like the cell core, Golgi apparatus, mitochondria, and endoplasmic reticulum, it seemed less likely that BioSAXS could detect changes since so many structures of the yeast cells most likely stay intact when treated with antifungals. We wanted to investigate whether it is possible to detect ultrastructural changes in an eukaryote. Surprisingly, such ultrastructural changes could indeed be measured by BioSAXS using Flucytosine as an antifungal drug. To the best of our knowledge, this is the first study to show that BioSAXS can be used successfully to differentiate between untreated *C. albicans* cells and those treated with an antifungal compound. Based on this success, different antifungal peptides were used and all of them showed differentiating effects from the untreated cells. This is the first proof-of-principle study with an exciting outcome, however, this is the very beginning of the work, and various studies need to be performed to verify the data, expand on known antifungal drugs, and perform microscopy in parallel. These experiences are important for learning more about the potential applications, robustness, and limitations of this method. The final aim is to use BioSAXS as a method to develop novel antifungal drugs with new modes of action faster, cheaper and with less risk involved, see Figure 3.

### Limitation

This study is a small explorative one, that by its very nature has limitations. Here only one *C. albicans* strain and one commercial antifungal at one time point and one concentration was used. Further studies should include various strains of *C. albicans* and all commercially available antifungals used in a time- and concentration-dependent manner at a higher number of repeats. Another limitation is that for this study we did not perform microscopy or other techniques in parallel to correlate such data. In further studies, the establishment of correlation with traditional methods and BioSAXS should be performed.

## Conclusion

BioSAXS was very successful in detecting ultrastructural changes in bacteria and therefore provide data for the classification of modes of action (Rumancev et al., 2022). However, fungi are eukaryotes and therefore contain many different internal structures than bacteria. Will the ultrastructural changes induced by an antifungal substance be large enough to be detected? This proof of principle study shows that despite the eukaryote inherent structures BioSAXS could detect ultrastructural changes of a clinically used antifungal compound and five antifungal peptides. That opens up opportunities for further studies for verification of this application and consequently the use of BioSAXS to find urgently needed antifungals with new modes of action (Hultmark et al., 1982).

## Data Availability

The raw data supporting the conclusion of this article will be made available by the authors, without undue reservation.

## Supplementary material

SUPPLEMENTARY FIGURE S1 SAXS curves with error bars. Each sample was measured 20 times to obtain the SAXS curve. Errors are standard deviations; these errors were propagated to the PCA values. For better visualization, the errors have been removed from the main Figure 1.

## References

[B1] Almaaytah A. Albalas Q. (2014). Scorpion venom peptides with no disulfide bridges: A review. Peptides 51, 35–45. 10.1016/J.PEPTIDES.2013.10.021 24184590

[B2] Andreu D. Ubach J. Boman A. Wåhlin B. Wade D. Merrifield R. B. (1992). Shortened cecropin A-melittin hybrids. Significant size reduction retains potent antibiotic activity. FEBS Lett. 296, 190–194. 10.1016/0014-5793(92)80377-S 1733777

[B3] Arastehfar A. Boekhout T. Butler G. Buda De Cesare G. Dolk E. Gabaldón T. (2019). Recent trends in molecular diagnostics of yeast infections: From PCR to NGS. FEMS Microbiol. Rev. 43, 517–547. 10.1093/FEMSRE/FUZ015 31158289 PMC8038933

[B4] Ashby M. Petkova A. Gani J. Mikut R. Hilpert K. (2017). Use of peptide libraries for identification and optimization of novel antimicrobial peptides. Curr. Top. Med. Chem. 17, 537–553. 10.2174/1568026616666160713125555 27411326

[B5] Ashby M. Petkova A. Hilpert K. (2014). Cationic antimicrobial peptides as potential new therapeutic agents in neonates and children: A review. Curr. Opin. Infect. Dis. 27, 258–267. 10.1097/QCO.0000000000000057 24722240

[B7] Battista F. Oliva R. Vecchio P. D. Winter R. Petraccone L. (2021). Insights into the action mechanism of the antimicrobial peptide Lasioglossin III. Int. J. Mol. Sci. 22, 2857. 10.3390/IJMS22062857 33799744 PMC8001998

[B57] Bhattacharya S. Sae-Tia S. Fries B. C. (2020). Candidiasis and mechanisms of antifungal resistance. Antibiotics 9 (6), 312. 10.3390/ANTIBIOTICS9060312 32526921 PMC7345657

[B8] Boldon L. Laliberte F. Liu L. (2015). Review of the fundamental theories behind small angle X-ray scattering, molecular dynamics simulations, and relevant integrated application. Nano Rev. 6, 25661. 10.3402/NANO.V6.25661 25721341 PMC4342503

[B9] Byer A. S. Pei X. Patterson M. G. Ando N. (2023). Small-angle X-ray scattering studies of enzymes. Curr. Opin. Chem. Biol. 72, 102232. 10.1016/J.CBPA.2022.102232 36462455 PMC9992928

[B10] Cao L. Dai C. Li Z. Fan Z. Song Y. Wu Y. (2012). Antibacterial activity and mechanism of a scorpion venom peptide derivative *in vitro* and *in vivo* . PLoS One 7 (7), 40135. 10.1371/journal.pone.0040135 PMC3390344 22792229

[B11] Chetty T. Mhlongo J. T. Waddad A. Y. Albericio F. de la Torre B. G. (2022). Novel CA(1–7)M(2–9) analogs: Synthesis, characterization, and antibacterial evaluation. ACS Med. Chem. Lett. 13 (8), 1370–1377. 10.1021/acsmedchemlett.2c00270 35978681 PMC9377004

[B12] Cerovsky V. Budesinsky M. Hovorka O. Cvacka J. Voburka Z. Slaninová J. (2009). Lasioglossins: Three novel antimicrobial peptides from the venom of the eusocial bee *Lasioglossum laticeps* (hymenoptera: Halictidae). ChemBioChem 10, 2089–2099. 10.1002/cbic.200900133 19591185

[B13] Edlund C. Hedberg M. Engström Å. Flock J. I. Wade D. (1998). Antianaerobic activity of a cecropin---melittin peptide. Clin. Microbiol. Infect. 4, 181–185. 10.1111/J.1469-0691.1998.TB00666.X 11864323

[B14] Franke D. Svergun D. I. (2020). Synchrotron small-angle X-ray scattering on biological macromolecules in solution. Synchrotron Light Sources free. Lasers Accel. Phys. Instrum. Sci. Appl. 2020, 1645–1672. 10.1007/978-3-030-23201-6_34/COVER

[B15] Gawde U. Chakraborty S. Waghu F. H. Barai R. S. Khanderkar A. Indraguru R. (2023). CAMPR4: A database of natural and synthetic antimicrobial peptides. Nucleic Acids Res. 51, D377–D383. 10.1093/NAR/GKAC933 36370097 PMC9825550

[B16] Grimsey E. Collis D. W. P. Mikut R. Hilpert K. (2020). The effect of lipidation and glycosylation on short cationic antimicrobial peptides. Biochim. Biophys. Acta - Biomembr. 1862, 183195, 10.1016/j.bbamem.2020.183195 32130974

[B17] Hilpert K. Elliott M. R. Volkmer-Engert R. Henklein P. Donini O. Zhou Q. (2006). Sequence requirements and an optimization strategy for short antimicrobial peptides. Chem. Biol. 13, 1101–1107. 10.1016/j.chembiol.2006.08.014 17052614

[B18] Hilpert K. Gani J. Rumancev C. Simpson N. Lopez-Perez P. M. Garamus V. M. (2021). Rational designed hybrid peptides show up to a 6-fold increase in antimicrobial activity and demonstrate different ultrastructural changes as the parental peptides measured by BioSAXS. Front. Pharmacol. 1, 769739. 10.3389/FPHAR.2021.769739 PMC8711299 34966279

[B19] Hilpert K. McLeod B. Yu J. Elliott M. R. Rautenbach M. Ruden S. (2010). Short cationic antimicrobial peptides interact with ATP. Antimicrob. Agents Chemother. 54, 4480–4483. 10.1128/AAC.01664-09 20660668 PMC2944583

[B20] Hilpert K. Volkmer-Engert R. Walter T. Hancock R. E. W. (2005). High-throughput generation of small antibacterial peptides with improved activity. Nat. Biotechnol. 23, 1008–1012. 10.1038/nbt1113 16041366

[B21] Hirai Y. Yasuhara T. Yoshida H. Nakajima T. (1981). A new mast cell degranulating peptide, mastoparan-M, in the venom of the hornet Vespa mandarinia. Biomed. Res. 2, 447–449. 10.2220/BIOMEDRES.2.447

[B22] Honecker D. Bersweiler M. Erokhin S. Berkov D. Chesnel K. Venero D. A. (2022). Using small-angle scattering to guide functional magnetic nanoparticle design. Nanoscale Adv. 4, 1026–1059. 10.1039/D1NA00482D 36131777 PMC9417585

[B23] Hultmark D. Engström Å. Bennich H. Kapur R. Boman H. G. (1982). Insect immunity: Isolation and structure of cecropin D and four minor antibacterial components from cecropia pupae. Eur. J. Biochem. 127, 207–217. 10.1111/J.1432-1033.1982.TB06857.X 7140755

[B24] Hultmark D. Steiner H. Rasmuson T. Boman H. G. (1980). Insect immunity. Purification and properties of three inducible bactericidal proteins from hemolymph of immunized pupae of Hyalophora cecropia. Eur. J. Biochem. 106, 7–16. 10.1111/J.1432-1033.1980.TB05991.X 7341234

[B26] Kang X. Dong F. Shi C. Liu S. Sun J. Chen J. (2019). DRAMP 2.0, an updated data repository of antimicrobial peptides. Sci. Data 6 (148), 1–10. 10.1038/s41597-019-0154-y 31409791 PMC6692298

[B58] Kean R. Ramage G. (2019). Combined antifungal resistance and biofilm tolerance: the global threat of Candida auris. MSphere 4 (4). 10.1128/msphere.00458-19 PMC6669339 31366705

[B27] Knappe D. Ruden S. Langanke S. Tikkoo T. Ritzer J. Mikut R. (2016). Optimization of oncocin for antibacterial activity using a SPOT synthesis approach: Extending the pathogen spectrum to *Staphylococcus aureus* . Amino Acids 48, 269–280. 10.1007/s00726-015-2082-2 26334348

[B28] Kodedová M. Sychrová H. (2016). High-throughput fluorescence screening assay for the identification and comparison of antimicrobial peptides’ activity on various yeast species. J. Biotechnol. 233, 26–33. 10.1016/J.JBIOTEC.2016.06.023 27369550

[B29] Koehbach J. Gani J. Hilpert K. Craik D. J. (2021). Comparison of a short linear antimicrobial peptide with its di-sulfide-cyclized and cyclotide-grafted variants against clinically relevant pathogens. Microorganism 9, 1249. 10.3390/MICROORGANISMS9061249 PMC8228819 34201398

[B31] López-Pérez P. M. P. M. Grimsey E. Bourne L. Mikut R. Hilpert K. (2017). Screening and optimizing antimicrobial peptides by using SPOT-synthesis. Front. Chem. 5, 25. 10.3389/fchem.2017.00025 28447030 PMC5388751

[B32] Mania D. Hilpert K. Ruden S. Fischer R. Takeshita N. (2010). Screening for antifungal peptides and their modes of action in Aspergillus nidulans. Appl. Environ. Microbiol. 76, 7102–7108. 10.1128/AEM.01560-10 20833782 PMC2976221

[B33] Mardirossian M. Pérébaskine N. Benincasa M. Gambato S. Hofmann S. Huter P. (2018). The dolphin proline-rich antimicrobial peptide Tur1A inhibits protein synthesis by targeting the bacterial ribosome. Cell Chem. Biol. 25, 530–539.e7. 10.1016/j.chembiol.2018.02.004 29526712 PMC6219704

[B34] Mardirossian M. Sola R. Beckert B. Collis D. W. P. Di Stasi A. Armas F. (2019). Proline-rich peptides with improved antimicrobial activity against *E. coli*, *K. pneumoniae* and A. baumannii. ChemMedChem 14, 2025–2033. 10.1002/cmdc.201900465 31692278 PMC6973051

[B35] Mardirossian M. Sola R. Beckert B. Valencic E. Collis D. W. P. Borišek J. (2020). Peptide inhibitors of bacterial protein synthesis with broad spectrum and SbmA-independent bactericidal activity against clinical pathogens. J. Med. Chem. 63, 9590–9602. 10.1021/acs.jmedchem.0c00665 32787108

[B36] Mikut R. Bartschat A. Doneit W. González∼Ordiano J. A. Schott B. Stegmaier J. (2017). The MATLAB Toolbox SciXMiner: User’s manual and programmer’s guide. https://arxiv.org/abs/1704.03298.

[B37] Mikut R. (2010). Computer-based analysis, visualization, and interpretation of antimicrobial peptide activities. Methods Mol. Biol. 618, 287–299. 10.1007/978-1-60761-594-1_18 20094871

[B38] Mikut R. Ruden S. Reischl M. Breitling F. Volkmer R. Hilpert K. (2016). Improving short antimicrobial peptides despite elusive rules for activity. Biochim. Biophys. Acta - Biomembr. 1858, 1024, 1033. 10.1016/j.bbamem.2015.12.013 26687790

[B59] Murray C. J. Ikuta K. S. Sharara F. Swetschinski L. Robles Aguilar G. Gray A. (2022). Global burden of bacterial antimicrobial resistance in 2019: a systematic analysis. The Lancet 399 (10325), 629–655. 10.1016/S0140-6736(21)02724-0/ATTACHMENT/2CA9AF18-3728-4446-B9C7-CCE47BAFEB4F/MMC1.PDF PMC8841637 35065702

[B39] Pirtskhalava M. Amstrong A. A. Grigolava M. Chubinidze M. Alimbarashvili E. Vishnepolsky B. (2021). DBAASP v3: Database of antimicrobial/cytotoxic activity and structure of peptides as a resource for development of new therapeutics. Nucleic Acids Res. 49, D288–D297. 10.1093/NAR/GKAA991 33151284 PMC7778994

[B40] Raghuraman H. Chattopadhyay A. (2007). Melittin: A membrane-active peptide with diverse functions. Biosci. Rep. 27, 189–223. 10.1007/S10540-006-9030-Z 17139559

[B41] Rumancev C. Rosenhahn A. Hilpert K. (2022). BioSAXS -an emerging method to accelerate, enrich and de-risk antimicrobial drug development. Front. Pharmacol. 1, 947005. 10.3389/FPHAR.2022.947005 PMC9445215 36081947

[B42] Sartori B. Marmiroli B. (2022). Tailoring lipid-based drug delivery nanosystems by synchrotron small angle X-ray scattering. Pharmaceutics 14, 2704. 10.3390/PHARMACEUTICS14122704 36559196 PMC9781362

[B43] Semeraro E. F. Marx L. Mandl J. Letofsky-Papst I. Mayrhofer C. Frewein M. P. (2022). Lactoferricins impair the cytosolic membrane of *Escherichia coli* within a few seconds and accumulate inside the cell. Elife 11, e72850. 10.7554/ELIFE.72850 35670565 PMC9352351

[B44] Slaninová J. Putnová H. Borovičková L. Šácha P. Čeřovský V. Monincová L. (2011). The antifungal effect of peptides from hymenoptera venom and their analogs. Cent. Eur. J. Biol. 6, 150–159. 10.2478/s11535-010-0111-4

[B45] Sun Y. Li X. Chen R. Liu F. Wei S. (2022). Recent advances in structural characterization of biomacromolecules in foods via small-angle X-ray scattering. Front. Nutr. 9, 1039762. 10.3389/FNUT.2022.1039762 36466419 PMC9714470

[B46] Vermes A. Guchelaar H. J. Dankert J. (2000). Flucytosine: A review of its pharmacology, clinical indications, pharmacokinetics, toxicity and drug interactions. J. Antimicrob. Chemother. 46, 171–179. 10.1093/JAC/46.2.171 10933638

[B47] Von Gundlach A. Ashby M. P. Gani J. Lopez-Perez P. M. Cookson A. R. Huws S. A. (2019). BioSAXS measurements reveal that two antimicrobial peptides induce similar molecular changes in Gram-negative and Gram-positive bacteria. Front. Pharmacol. 10, 1127. 10.3389/fphar.2019.01127 31616307 PMC6775230

[B48] Von Gundlach A. R. Garamus V. M. Gorniak T. Davies H. A. Reischl M. Mikut R. (2016a). Small angle X-ray scattering as a high-throughput method to classify antimicrobial modes of action. Biochim. Biophys. Acta - Biomembr. 1858, 918, 925. 10.1016/j.bbamem.2015.12.022 26730877

[B49] Von Gundlach A. R. R. Garamus V. M. M. Willey T. M. M. Ilavsky J. Hilpert K. Rosenhahn A. (2016b). Use of small-angle X-ray scattering to resolve intracellular structure changes of *Escherichia coli* cells induced by antibiotic treatment. J. Appl. Crystallogr. 49, 2210–2216. 10.1107/S1600576716018562 27980516 PMC5139998

[B50] Vrablikova A. Czernekova L. Cahlikova R. Novy Z. Petrik M. Imran S. (2017). Lasioglossins LLIII affect the morphogenesis of Candida albicans and reduces the duration of experimental vaginal candidiasis in mice. Microbiol. Immunol. 61, 474–481. 10.1111/1348-0421.12538 28892177

[B51] Wang G. Li X. Wang Z. (2016). APD3: The antimicrobial peptide database as a tool for research and education. Nucleic Acids Res. 44, D1087–D1093. 10.1093/nar/gkv1278 26602694 PMC4702905

[B52] Wiegand I. Hilpert K. Hancock R. E. W. (2008). Agar and broth dilution methods to determine the minimal inhibitory concentration (MIC) of antimicrobial substances. Nat. Protoc. 3, 163–175. 10.1038/nprot.2007.521 18274517

[B53] Wu T. M. Chou T. C. Ding Y. A. Li M. L. (1999). Stimulation of TNF-alpha, IL-1beta and nitrite release from mouse cultured spleen cells and lavaged peritoneal cells by mastoparan M. Immunol. Cell Biol. 77, 476–482. 10.1046/J.1440-1711.1999.00847.X 10571667

[B54] Wu T. M. Li M. L. (1999). The cytolytic action of all-D mastoparan M on tumor cell lines. Int. J. Tissue React. 21, 35–42. 10568222

[B56] Zeng X. C. Wang S. X. Zhu Y. Zhu S. Y. Li W. X. (2004). Identification and functional characterization of novel scorpion venom peptides with no disulfide bridge from Buthus martensii Karsch. Peptides 25, 143–150. 10.1016/J.PEPTIDES.2003.12.003 15062994

