# Supplementary material for: Can BioSAXS detect ultrastructural changes of antifungal compounds in Candida albicans?–an exploratory study
Source: Front Pharmacol. 2023 Jul 18;14:1141785. doi: 10.3389/fphar.2023.1141785 (PMC10393279; doi:10.3389/fphar.2023.1141785)
Supplement: Supplementary file 1 [file Image1.pdf]

Supplemental material

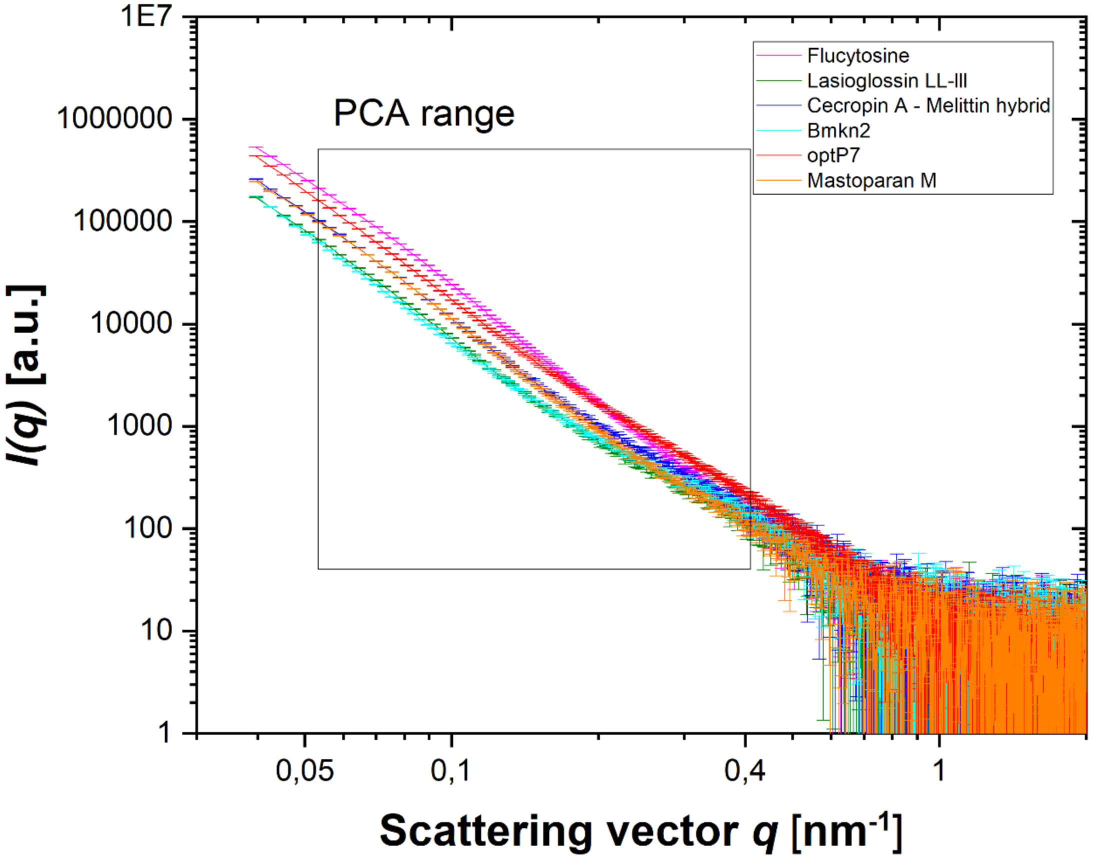

Supplementary Figure 1: SAXS curves with error bars. Each sample was measured 20 times to obtain the SAXS curve. Errors are standard deviations; this error were propagated to the PCA values. For better visualization the errors have been removed from the main Figure 1.
